# Body composition assessment for risk stratification of hepatic steatosis in patients with type 2 diabetes and MASLD undergoing osteoporosis screening

**DOI:** 10.3389/fendo.2026.1901793

**Published:** 2026-08-14

**Authors:** Clelia Asero, Cecilia Oliveri, Maria Stella Franzè, Giorgio Basile, Antonino Catalano, Irene Cacciola

**Affiliations:** 1 Division of Medicine and Hepatology, University Hospital of Messina, Messina, Italy; 2 Department of Clinical and Experimental Medicine, University of Messina, Messina, Italy; 3 Geriatric Unit, University Hospital of Messina, Messina, Italy; 4 Department of Biomedical and Dental Science and Morphofunctional Imaging, University of Messina, Messina, Italy

**Keywords:** body composition, dual energy X ray absorptiometry, fat mass, MASLD, obesity, type 2 diabetes

## Abstract

**Background and aims:**

Metabolic dysfunction-associated steatotic liver disease (MASLD) is the most common cause of chronic liver disease worldwide. Type 2 diabetes (T2D) and obesity are major risk factors for its development and progression. Body mass index (BMI), although widely used in clinical practice, fails to adequately reflect individual differences in body composition and fat distribution. This study evaluated the association of adiposity and reduced muscle mass – measured by dual-energy X-ray absorptiometry (DXA) – with hepatic steatosis severity in patients with MASLD and T2D.

**Methods:**

Consecutive patients with MASLD and T2D were recruited at a tertiary hepatology centre. Body composition was assessed by DXA. Adiposity was defined using fat mass percentage, with values >25% in men and >35% in women indicating obesity. Muscle mass was assessed using the appendicular lean mass/height^2^ (ALM/height²) index, with pathological values defined as <7.0 kg/m^2^ in men and <5.5 kg/m^2^ in women. Hepatic steatosis and fibrosis were evaluated by controlled attenuation parameter (CAP) and liver stiffness measurement (LSM), respectively. Statistical analyses included Spearman’s rank correlation, Chi Square test, logistic regression, and receiver operating characteristic (ROC) curve analyses.

**Results:**

Ninety-seven patients (median age 61 years) were enrolled. DXA identified a pathological fat mass percentage in 97.9% of patients, whereas 46.4% met the BMI-based criteria for obesity. At multivariate analysis, fat mass percentage and pathological ALM/height^2^ were independently related with severe hepatic steatosis (CAP ≥280 dB/m; OR 1.100, *p* = 0.011; OR 0.292, *p* = 0.013, respectively). ROC curve analysis identified specific cut-offs for fat mass percentage (38.65%, area under the curve [AUC] of 0.636) and ALM/height^2^ index (6.7 kg/m^2^, AUC of 0.611) for detecting severe liver steatosis. A model combining both cut-offs yielded an AUC of 0.715 (95% C.I. 0.610 – 0.820, *p* = 0.001).

**Conclusions:**

In patients with MASLD and T2D, DXA-derived body composition measures may complement conventional anthropometric assessment and help identify individuals with severe hepatic steatosis.

## 1 Introduction

Metabolic dysfunction-associated steatotic liver disease (MASLD) is the leading cause of chronic liver disease worldwide (1). Epidemiological data from 2016–2019 indicate a global adult prevalence of approximately 38%, which is projected to increase to 55.4% by 2040 (2). Current international consensus define MASLD as a chronic condition characterized by hepatic steatosis in the presence of at least one cardiometabolic risk factor (1), thereby reinforcing its role as the hepatic manifestation of Metabolic Syndrome.

Among MASLD-associated comorbidities, type 2 diabetes (T2D) and obesity are major drivers of disease onset and progression. MASLD affects around 60-70% of individuals with T2D and up to 80% of those with obesity, contributing substantially to both liver-related and extrahepatic morbidity and mortality (3, 4). The pathogenesis of MASLD is complex and multifactorial. Key mechanisms include increased hepatic uptake of free fatty acids from dietary sources and adipose tissue, enhanced hepatic *de novo* lipogenesis, reduced fatty acids oxidation, and impaired export of triglycerides through very-low-density lipoproteins. Chronic low-grade systemic inflammation arising from dysfunctional adipose tissue also contributes to hepatocellular injury and fibrogenesis (3). Notably, the coexistence of T2D and central obesity markedly accelerates disease progression and is strongly associated with adverse liver-related outcomes (5).

Obesity plays a crucial role in MASLD pathophysiology. It is a chronic disease and a major public health challenge, with projections suggesting that up to 3.8 billion individuals may be affected by 2050 (6). In clinical practice, obesity is commonly assessed using body mass index (BMI), with a diagnostic threshold of ≥30 kg/m^2^ (7). In individuals with obesity, excessive and dysfunctional adipose tissue promotes insulin-resistance and increased free fatty acids flux to the liver, ultimately leading to hepatic lipid accumulation and metabolic dysfunction (6). However, beyond excess body weight per se, body fat distribution critically influences both the development and severity of MASLD. Individuals with normal BMI may nonetheless exhibit increased visceral adiposity, a phenotype associated with hepatic steatosis, insulin resistance, and high cardiovascular risk, a condition commonly referred as “lean MASLD” (2). Both lean and non-lean MASLD phenotypes are characterized by visceral and ectopic fat accumulation, as well as dysregulated adipokine secretion, including reduced adiponectin levels and increased leptin, tumour necrosis factor-α and interleukin-6 concentrations. These changes promote hepatic inflammation and fibrogenesis.

In parallel, sarcopenia, defined as the progressive loss of skeletal muscle mass and function (8), has emerged as an additional factor associated with the development and progression of MASLD (9). The relationship between sarcopenia and MASLD is complex and bidirectional, involving insulin resistance, metabolic dysregulation and chronic inflammation. These mechanisms provide a rationale for structured physical activity interventions to preserve muscle mass and function, while improving metabolic homeostasis (1).

In this context, BMI, although widely used to assess nutritional status, has well-recognized limitations, particularly its inability to discriminate between fat mass and lean mass and to adequately characterize regional adipose tissue distribution. These shortcomings are clinically relevant, as visceral adiposity exhibits a stronger association with metabolic complications than overall body weight (10).

Several techniques can complement anthropometric measurements in the assessment of body composition, including bioelectrical impedance approaches, computerized tomography (CT), ultrasound (US), and dual-energy X-ray absorptiometry (DXA) (11). Among these methods, DXA offers a favourable balance between accuracy, reproducibility, low radiation exposure, and feasibility in routine clinical practice (12, 13).

The aim of the present study was to investigate the relationship between body composition, by considering adiposity and reduced muscle mass, and steatosis severity in patients with MASLD and T2D.

## 2 Materials and methods

### 2.1 Study population selection and exclusion criteria

This cross-sectional study included consecutive patients with MASLD and T2D who attended the Division of Medicine and Hepatology of the University Hospital of Messina between February and October 2024. All participants underwent DXA for osteoporosis screening according to international guidelines for T2D management, together with a concurrent assessment of body composition (14, 15).

Exclusion criteria comprised: pre-menopausal status in women and age <50 years in men; presence of active malignancies; other causes of chronic liver disease, including viral hepatitis, autoimmune and cholestatic liver disease (1); decompensated cirrhosis, defined by the presence of ascites, hepatic encephalopathy, gastrointestinal bleeding, and/or a Child Pugh Score >B (16); harmful alcohol intake or daily consumption >50 g in women and >60 g in men (1, 17); conditions potentially affecting body composition assessment, such as dehydration, oedema, recent intense physical activity before the examination, caffeine or food intake prior to DXA assessment (18).

All participants provided written informed consent. Clinical, biochemical, and imaging data were collected at the enrolment and recorded in a digitalized database. The study was approved by the Ethics Committee at the University Hospital of Messina (protocol number: 2024/7) and conducted in accordance with the Declaration of Helsinki.

### 2.2 Clinical, anthropometric and laboratory data

Clinical variables included age, sex, and smoking habit. A comprehensive medical history was obtained, including dyslipidaemia and arterial hypertension, related pharmacological treatments, duration of T2D and liver disease, and presence of microvascular and macrovascular diabetic complications.

Liver disease was classified as either liver cirrhosis or chronic liver disease (CLD) based on histological findings (when available), together with ultrasonographic and biochemical criteria, liver stiffness measurement (LSM), and upper gastrointestinal endoscopy findings (19). All patients underwent liver ultrasound (US) and vibration-controlled transient elastography with controlled attenuation parameter (CAP) assessment. Hepatic steatosis on US was defined by increased hepatic echogenicity compared with the right kidney associated with a progressive attenuation of vascular and diaphragmatic structures. LSM was performed after overnight fasting, using M or XL probes according to manufacturer recommendations. Hepatic steatosis was defined as CAP ≥ 248 dB/m (mild ≥ 248 dB/m, moderate ≥ 268 dB/m, severe ≥ 280 dB/m), whereas liver stiffness ≥ 8 kPa was considered indicative of CLD (1). Each examination included at least ten valid measurements, with an interquartile range (IQR)/median ratio <30%, ensuring adequate examination quality.

Anthropometric measurements included BMI, waist circumference (WC), calf circumference (CC), and visceral adiposity index (VAI). According to international guidelines, overweight was defined as BMI ≥25 kg/m^2^, whereas obesity was defined as BMI ≥30 kg/m^2^ and/or WC >94 cm in men and >80 cm in women (1). VAI was calculated using sex-specific formulas (20).

Sarcopenia was assessed through the evaluation of both muscle mass and muscle strength. Muscle mass was estimated using CC, measured at the widest point of the lower leg, and values <31 cm were considered indicative of reduced muscle mass. Muscle strength was assessed using handgrip strength measured with a Jamar dynamometer, with established cut-off for low muscle strength of <27 kg in men and <16 kg in women (8). Physical activity levels were evaluated using the International Physical Activity Questionnaire (IPAQ), which categorized physical activity into three levels of intensity: low, moderate, and high (21).

Biochemical parameters included serum alanine aminotransferase (ALT), aspartate aminotransferase (AST), gamma-glutamyl transferase (GGT), alkaline phosphatase (ALP), total bilirubin, albumin, gamma globulins, international normalized ratio (INR), platelet (PLT) count, fasting plasma glucose, glycated haemoglobin (HbA1c), total cholesterol, high-density lipoprotein cholesterol (HDL-c), low-density lipoprotein cholesterol (LDL-c), triglycerides, and serum creatinine. Estimated Glomerular Filtration Rate (eGFR) was calculated applying the chronic kidney disease epidemiology consortium estimated glomerular filtration rate (CKD-EPI) 2021 race-free equation. The Triglyceride-Glucose index (TyG) was assessed using the following formula: log [fasting triglycerides (mg/dL) * fasting glucose (mg/dL)/2].

### 2.3 Body composition assessment

Body composition was assessed using DXA densitometer (Hologic Discovery Wi) according to a standardized protocol. The densitometer was calibrated daily according to the manufacturer’s recommendations. All examinations were performed at a controlled room temperature (21 °C), with participants in the supine position after an overnight fast of at least eight hours, to minimize variability related to hydration status and peripheral blood flow.

Adiposity was evaluated using fat mass percentage and android-to-gynoid fat mass ratio. Fat mass percentage was calculated as fat mass (kg)/total mass (kg)*100. Obesity was defined according to World Health Organization (WHO) criteria as body fat mass percentage >25% in men and >35% in women (22). Fat distribution was evaluated using the android-to-gynoid fat mass ratio. Values >1.0 in men and >0.8 in women were considered pathological, reflecting an “apple shape” fat distribution pattern (22). Reduced muscle mass was identified using the appendicular lean mass/height^2^ (ALM/height²) index expressed in kg/m^2^. According to current international guidelines, pathological values were identified as <7.0 kg/m^2^ in men and <5.5 kg/m^2^ in women (8, 22).

### 2.4 Statistical analysis

Continuous variables were expressed as median and interquartile range (IQR), while categorical variables were reported as number and percentages. Data distribution normality was assessed using the Kolmogorov-Smirnov test. Correlations between continuous variables were evaluated using Spearman’s rank correlation coefficient because of non-normal data distribution. Groups were compared using the Kruskal–Wallis test for continuous variables and the chi-square test for categorical variables.

Univariate logistic regression analyses were first performed to identify factors independently associated with severe hepatic steatosis. Variables associated with the outcome at p value < 0.05 in univariate analysis were subsequently included in a multivariate logistic regression model. To limit the model complexity and reduce conceptual redundancy among closely related anthropometric measures, one variable per domain (adiposity, muscle mass, and clinical factors) was selected for inclusion in the final model. Regression coefficients were estimated using maximum likelihood estimation, and results were reported as odds ratios (OR) with 95% confidence intervals (C.I.). Receiver operating characteristic (ROC) curve analysis was performed to evaluate the discriminative ability of body composition parameters for CAP ≥280 dB/m, and optimal cut-off values were identified using the Youden index. Predicted probabilities derived from the multivariate logistic regression model were calculated for each subject and used to generate ROC curves, enabling assessment of the overall discriminative performance of the combined model. A p-value <0.05 was considered statistically significant. Areas under correlated ROC curves (AUCs) were compared pairwise using DeLong’s test in R, version 4.2.0 (R Foundation for Statistical Computing, Vienna, Austria). All analyses than DeLong’s Test were performed using SPSS, version 25.0 for Windows package. No formal *a priori* sample size calculation was performed, as this was an exploratory single-centre study based on a consecutive cohort of patients with MASLD and T2D undergoing DXA assessment. A sensitivity power analysis was performed to determine the minimum AUC detectable against the null hypothesis value of 0.50, assuming a two-sided α level of 0.05 and 80% statistical power.

## 3 Results

### 3.1 Characteristics of the study population

Ninety-seven consecutive patients met the inclusion criteria and were enrolled in the study. Demographic, anthropometric, clinical, and instrumental characteristics of study population are summarized in **Table 1**.

**Table 1:** Demographic, anthropometric, clinical, biochemical and instrumental features of 97 patients with MASLD and T2D included in the study.

Variables	All patients (n = 97)
Age, years	61 (56 – 68)
Male sex, n	52 (53.6)
BMI, kg/m^2^	29.3 (26.5 – 32.9)
BMI >30 kg/m^2^	45 (46.4%)
Waist circumference, cm	102 (95 – 110)
Pathological waist circumference, n	86 (88.6)
Visceral Adiposity Index	11.5 (6.975 – 18.20)
Fat mass percentage, %	39.6 (34.72 – 46.77)
Pathological Total Fat, n	95 (97.9)
Pathological Android-to-Gynoid ratio, n	91 (93.8)
ALM/Height^2^, kg/m^2^	7 (5.65 – 7.93)
Pathological ALM/Height^2^, n	35 (36.1)
Handgrip strength, kg	36 (26 – 48.5)
Pathological handgrip strength, n	9 (9.27)
Calf circumference, cm	38 (32.5 – 40)
Calf circumference <31 cm, n	20 (20.6)
IPAQ categories, n	
- Low grade - Moderate grade - High grade	81 (83.5) 12 (12.4) 4 (4.1)
Liver cirrhosis, n	17 (17.5)
Liver Stiffness, kPa	7.3 (5.8 – 12.8)
CAP, dB/m	297 (253 – 325)
CAP ≥280 dB/m	62 (63.9)
AST, U/L	29 (21 – 39)
ALT, U/L	36 (22 – 48)
GGT, U/L	40 (24 – 80)
ALP, U/L	80 (60.75 – 106.5)
Bilirubin, mg/dL	0.7 (0.5 – 0.84)
Albumin, g/dL	4.13 (3.8 – 4.37)
Gamma-globulins, g/dL	1 (0.86 – 1.23)
INR	1.03 (0.97 – 1.14)
PLT, mmc	218 (172 – 255)
Fasting glucose, mg/dL	117 (101 – 138)
HbA1c, %	6.45 (5.9 – 7.1)
Total cholesterol, mg/dL	142 (122 – 171)
LDL-c, mg/dL	77.5 (53.2 – 102.8)
Triglycerides, mg/dL	129 (88.5 – 186)
TyG	4.79 (4.61 – 5.03)
CKD_EPI eGFR, ml/min/1.73 m^2^	86.8 (75.35 – 96.25)
Lipid lowering therapy, n	79 (81.4%)
LDL-c target for cardiovascular risk, n	44 (45.4)
Glucose lowering therapy, n - Insulin, n - Metformin, n - GLP1Ra, n - SGLT2i, n	88 (90.7) 18 (18.6) 78 (80.4) 37 (38.1) 27 (27.8)
Hypertension, n	78 (80.4)
Smoking habit, n	13 (13.4)
Liver disease, years	4 (2 – 8)
Type 2 diabetes, years	10 (3 – 13)

BMI, body mass index; ALM/Height^2^, Appendicular Lean Mass Height^2^ index; IPAQ, International Physical Activity Questionnaire; CAP, controlled attenuation parameter; ALT, alanine aminotransferase; AST, aspartate aminotransferase; GGT, gamma-glutamyl transferase; ALP, alkaline phosphatase; INR, international normalized ratio; PLT, platelets; HbA1c, glycated haemoglobin; TyG, Triglycerides Glucose Index; HDL-c, high-density lipoprotein cholesterol; LDL-c, low-density lipoprotein cholesterol; CKD-EPI eGFR, chronic kidney disease epidemiology consortium estimated glomerular filtration rate; GLP1Ra, Glucagon-Like Peptide-1 Receptor Agonist; SGLT2i, Sodium-Glucose Co-Transporter-2 Inhibitor.

Numerical variables are expressed as number and percentage (categorical) or mean and interquartile range (continuous).

At enrolment, median liver stiffness value was 7.3 kPa (IQR 5.8 – 12.8 kPa), and median CAP value was 297 dB/m (IQR 253–325 dB/m). Severe steatosis was observed in 62 patients (63.9%). Median aminotransferase levels were within the upper normal limit, and laboratory parameters reflecting liver function – including albumin, gamma-globulins, platelet count, INR, and bilirubin – were also within normal limits. Seventeen of the 97 patients (17.5%) had compensated liver cirrhosis. MASLD was histologically confirmed in 14 patients (14.4%).

Regarding metabolic comorbidities, the median HbA1c value was 6.45% (IQR 5.9 – 7.1%), indicating overall adequate glycaemic control despite elevated TyG index values (median 4.79, IQR 4.61 – 5.03). Microvascular complications of T2D were observed in 12 patients (12.4%), whereas macrovascular complication occurred in 17 patients (17.52%). Median LDL-cholesterol (LDL-c) was 77.5 mg/dL (IQR 53.2 – 102.8 mg/dL), although only 44 patients (45.4%) achieved the LDL-c target recommended by international guidelines according to individual cardiovascular risk (23). Arterial hypertension was present in 78 patients (80.4%), a higher proportion than the 40–60% reported in general MASLD populations (24), and 13 (13.4%) were active smokers. According to IPAQ categories, 81 patients (83.5%) reported low levels of physical activity, 12 (12.4%) moderate levels, and 4 (4.1%) high levels of physical activity.

Obesity was identified in 45 patients (46.4%) according to BMI criteria and in 86 patients (88.6%) based on waist circumference. DXA assessment revealed a pathological fat mass percentage in 95 patients (97.9%) and an abnormal android-to-gynoid fat mass ratio in 91 patients (93.8%). Chi Square Test showed a statistically significant difference between fat mass percentage and BMI values in detecting the presence of obesity in our population (p < 0.001), while android-to-gynoid fat mass ratio and waist circumference didn’t differ significantly in identifying it (p = 0.28). Twenty patients (20.6%) had a CC <31 cm, while nine (9.27%) showed reduced handgrip strength. Low ALM/height² was identified by DXA in 35 patients (36.1%) When comparing calf circumference and ALM/height^2^ index capacity in detecting reduced muscle mass, Chi Square Test showed statistically significant differences in the two methods applied for diagnosis (p = 0.016).

### 3.2 Body composition analysis and liver steatosis

Spearman’s rank correlation analysis was performed to evaluate relationships between anthropometric variables, DXA-derived body composition parameters, and hepatic steatosis severity based on CAP values.

Regarding the relationship between conventional anthropometric measures and DXA-derived indices, BMI showed a moderate positive correlation with fat mass percentage (Rho = 0.332, p = 0.001), whereas no significant correlation was observed with android-to-gynoid fat mass ratio (Rho = 0.002, p = 0.964). BMI was strongly correlated with waist circumference (Rho = 0.692, p < 0.001). Waist circumference did not correlate significantly with either fat mass percentage (Rho = 0.076, p = 0.460) or android-to-gynoid fat mass ratio (Rho = -0.099, p = 0.336).

CAP was weakly but significantly correlated with BMI (Rho = 0.274, p = 0.007), WC (Rho = 0.283, p = 0.005), VAI (Rho = 0.286, p = 0.005), and fat mass percentage (Rho = 0.245, p = 0.016). The android-to-gynoid fat mass ratio was not significantly correlated with CAP, whether analysed as a continuous or categorical variable (rho = 0.030, *p* = 0.772). No significant correlations with CAP were observed for the TyG index (rho = 0.200, *p* = 0.510), LDL-c (rho = 0.137, *p* = 0.182), or HbA1c (rho = 0.152, *p* = 0.138).

ALM/height² index, considered as a categorical variable – distinguishing patients with pathological versus normal values – showed a significant negative correlation with CAP (Rho = −0.276, p = 0.006), indicating that reduced appendicular lean mass was associated with higher CAP values.

**Figure 1:**
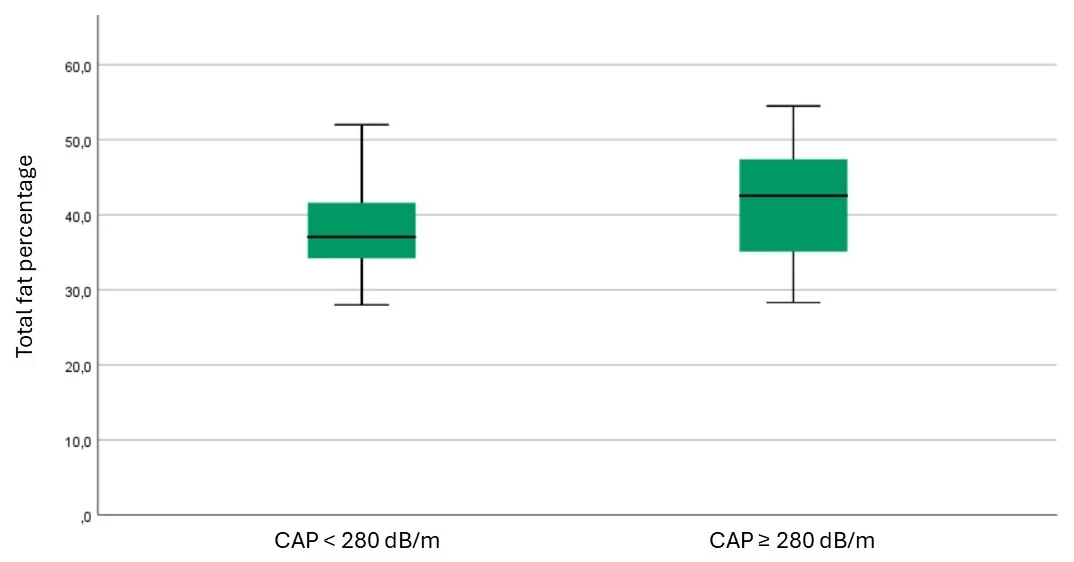
Boxplot showing the distribution of fat mass percentage according to hepatic steatosis severity stratified by CAP values (<280 dB/m vs ≥280 dB/m). Patients with severe hepatic steatosis (CAP ≥280 dB/m) showed higher fat mass percentage values compared with those with CAP <280 dB/m. The central line represents the median, the box indicates the interquartile range, and whiskers represent the minimum and maximum values. On the X-axis CAP values; the Y-axis represents fat mass percentage. CAP, Controlled Attenuation Parameter.

When focusing specifically on severe hepatic steatosis (CAP ≥ 280 dB/m), both fat mass percentage (Rho = 0.225, p = 0.027) and pathological ALM/height² index (Rho = −0.252, p = 0.013) remained significantly correlated with CAP, confirming the relevance of DXA-derived body composition parameters in identifying patients with the more advanced steatosis (**Figure 1**).

In contrast, none of BMI, waist circumference, VAI, android-to-gynoid fat mass ratio, fat mass percentage, ALM/height² and TyG index, showed significant correlations with liver stiffness values, either when analysed as continuous or categorical variables.

Logistic regression analyses were therefore performed to identify variables independently associated with severe steatosis and liver stiffness >8 kPa.

In the univariate regression analysis performed for CAP ≥ 280 dB/m, significant associations were observed for BMI (OR 1.154, 95% C.I. 1.031 – 1.290, p = 0.012), WC (OR 1.043, 95% C.I. 1.007 – 1.081, p = 0.021), fat mass percentage (OR 1.078, 95% C.I. 1.009 – 1.152, p = 0.027), pathological ALM/height^2^ index (OR 0.293, 95% C.I. 0.122 – 0.703, p= 0.006), handgrip strength (OR 0.967, 95% C.I. 0.940 – 0.994, p = 0.017), CC (OR 1.043, 95% C.I. 1.007 – 1.081, p = 0.021), and arterial hypertension (OR 3.094, 95% C.I. 1.105 – 8.665, p = 0.032). Multivariate analysis, performed using DXA-derived parameters confirmed significant associations between severe steatosis and fat mass percentage (OR 1.100, 95% C.I. 1.022 – 1.184, p = 0.011) as well as pathological appendicular lean mass (OR 0.292, 95% C.I. 1.111 – 0.771, p = 0.013). In contrast, arterial hypertension was no longer significantly associated with severe steatosis (OR 2.785, 95% C.I. 0.914 – 8.489, p = 0.072) (**Table 2**).

**Table 2:** Univariate and multivariate regression analysis performed for severe steatosis (CAP ≥280 dB/m).

Severe steatosis	Univariate regression analysis			Multivariate regression analysis		
Variables	P value	OR	95% C.I.	P value	OR	95% C.I.
Age	0.518	0.983	0.932 – 1.036			
Male sex	0.075	2.181	0.925 – 5.143			
BMI, kg/m^2^	**0.012**	**1.154**	**1.031 – 1.290**	–	–	–
Waist circumference, cm	**0.021**	**1.043**	**1.007 – 1.081**	–	–	–
Fat mass percentage, %	**0.027**	**1.078**	**1.009 – 1.152**	**0.011**	**1.100**	**1.022 – 1.184**
Pathological Android/Gynoid ratio, n	0.848	0.789	0.071 – 8.810			
ALM/Height^2^, kg/m^2^	0.071	1.299	0.978 – 1.726			
Pathological ALM/Height^2^, n	**0.006**	**0.293**	**0.122 – 0.703**	**0.013**	**0.292**	**0.111 – 0.771**
Handgrip strength, kg	**0.017**	**0.967**	**0.940 – 0.994**	–	–	–
Calf circumference, cm	**0.021**	**1.043**	**1.007 – 1.081**	–	–	–
IPAQ categories	0.119	0.517	0.226 – 1.185			
AST, U/L	0.601	1.007	0.981 – 1.034			
ALT, U/L	0.399	1.009	0.989 – 1.029			
GGT, U/L	0.498	0.998	0.993 – 1.003			
ALP, U/L	0.408	0.997	0.990 – 1.004			
Bilirubin, mg/dL	0.177	0.509	0.191 – 1.356			
Albumin, g/dL	0.359	0.694	0.319 – 1.514			
Gamma-globulin, g/dL	0.183	3.025	0.594 – 15.416			
INR	0.302	0.24	0.016 – 3.613			
PLT, mmc	0.149	1.005	0.998 – 1.011			
Fasting glucose, mg/dL	0.969	1.000	0.986 – 1.014			
HbA1c, %	0.466	1.156	0.783 – 1.707			
Total cholesterol, mg/dL	0.465	1.004	0.993 – 1.015			
HDL-cholesterol, mg/dL	0.286	0.981	0.947 – 1.016			
LDL-cholesterol, mg/dL	0.181	1.008	0.996 – 1.019			
Triglycerides, mg/dL	0.800	1.001	0.994 – 1.007			
TyG	0.499	1.667	0.379 – 7.329			
eGFR - CKD_EPI, ml/min/1.73 m^2^	0.708	1.005	0.978 – 1.034			
Lipid lowering therapy, n	0.784	1.159	0.404 – 3.325			
Insulin, n	0.784	0.863	0.301 – 2.475			
Metformin, n	0.543	1.374	0.494 – 3.822			
GLP1Ra, n	0.879	1.069	0.454 – 2.514			
SGLT2i, n	0.903	0.944	0.376 – 2.373			
**Hypertension, n**	**0.032**	**3.094**	**1.105 – 8.665**	0.072	2.785	0.914 – 8.489
Smoking habit, n	0.669	1.316	0.374 – 4.632			
Liver disease, years	0.540	0.974	0.896 – 1.059			
Type 2 diabetes, years	0.749	0.990	0.931 – 1.053			
Visceral Adiposity Index	0.626	1.009	0.973 – 1.047			

BMI, body mass index; ALM/Height^2^, Appendicular Lean Mass Height^2^ index; IPAQ, International Physical Activity Questionnaire; CAP, controlled attenuation parameter; ALT, alanine aminotransferase; AST, aspartate aminotransferase; GGT, gamma-glutamyl transferase; ALP, alkaline phosphatase; INR, international normalized ratio; PLT, platelets; HbA1c, glycated haemoglobin; TyG, Triglycerides Glucose Index; HDL-c, high-density lipoprotein cholesterol; LDL-c, low-density lipoprotein cholesterol; CKD-EPI eGFR, chronic kidney disease epidemiology consortium estimated glomerular filtration rate; GLP1Ra, Glucagon-Like Peptide-1 Receptor Agonist; SGLT2i, Sodium-Glucose Co-Transporter-2 Inhibitor.

Statistically significant variables are highlighted in bold type.

The same analyses were performed for liver stiffness values >8 kPa. In the univariate regression analysis, significant associations were observed for WC (OR 1.040, 95% C.I. 1.004 – 1.077, p = 0.030), AST (OR 1.044, 95% C.I. 1.010 – 1.078, p = 0.010), GGT (OR 1.026, 95% C.I. 1.012 – 1.041, p < 0.001), ALP (OR 1.015, 95% C.I. 1.003 – 1.026, p = 0.011), albumin (OR 0.291, 95% C.I. 0.111 – 0.768, p = 0.013), gamma-globulins (OR 5.651, 95% C.I. 1.161 – 27.514, p = 0.032), platelets (OR 0.991, 95% C.I. 0.984 – 0.998, p = 0.008), HbA1c (OR 1.593, 95% C.I. 1.055 – 2.405, p = 0.027), and insulin therapy (OR 3.097, 95% C.I. 1.053 – 9.110, p = 0.040). At multivariate regression analysis, WC (OR 1.102, 95% C.I. 1.031 – 1.179, p = 0.004), GGT (OR 1.035, 95% C.I. 1.013 – 1.058, p = 0.002) and platelet count (OR 0.988, 95% C.I. 0.977 – 1.000, p = 0.047) remained independently associated with liver stiffness values >8 kPa (**Table 3**).

**Table 3:** Univariate and multivariate regression analysis performed for liver stiffness > 8 kPa.

Stiffness > 8 kPa	Univariate regression analysis			Multivariate regression analysis		
Variables	P value	OR	95% C.I.	P value	OR	95% C.I.
Age	0.140	1.040	0.987 – 1.096			
Male sex	0.983	1.009	0.452 – 2.252			
BMI, kg/m^2^	0.501	1.032	0.941 – 1.133			
**Waist circumference, cm**	**0.030**	**1.040**	**1.004 – 1.077**	**0.004**	**1.102**	**1.031 – 1.179**
Fat mass percentage, %	0.939	1.002	0.945 – 1.063			
Android/Gynoid ratio, n	0.472	0.422	0.040 – 4.425			
ALM/Height^2^, kg/m^2^	0.825	0.973	0.766 – 1.237			
Pathological ALM/Height^2^, n	0.862	0.911	0.395 – 2.100			
Handgrip strength, kg	0.990	1.000	0.976 – 1.025			
Calf circumference, cm	0.558	1.016	0.964 – 1.070			
IPAQ categories	0.470	1.000	1.000 – 1.000			
**AST, U/L**	**0.010**	**1.044**	**1.010 – 1.078**	0.717	1.009	0.960 – 1.061
ALT, U/L	0.176	1.013	0.994 – 1.032			
**GGT, U/L**	**< 0.001**	**1.026**	**1.012 – 1.041**	**0.002**	**1.035**	**1.013 – 1.058**
**ALP, U/L**	**0.011**	**1.015**	**1.003 – 1.026**	0.578	1.004	0.991 – 1.017
Bilirubin, mg/dL	0.112	2.287	0.824 – 3.346			
**Albumin, g/dL**	**0.013**	**0.291**	**0.111 – 0.768**	0.779	0.820	0.205 – 3.285
**Gamma-globulins, g/dL**	**0.032**	**5.651**	**1.161 – 27.514**	0.093	9.286	0.692 – 124.649
INR	0.093	12.729	0.652 – 248.590			
**PLT, mmc**	**0.008**	**0.991**	**0.984 – 0.998**	**0.047**	**0.988**	**0.977 – 1.000**
Fasting glucose, mg/dL	0.124	1.011	0.997 – 1.025			
**HbA1c, %**	**0.027**	**1.593**	**1.055 – 2.405**	0.484	1.340	0.590 – 3.046
Total cholesterol, mg/dL	0.112	0.991	0.980 – 1.002			
HDL-cholesterol, mg/dL	0.115	0.972	0.938 – 1.007			
LDL-cholesterol, mg/dL	0.124	0.992	0.981 – 1.002			
Triglycerides, mg/dL	0.356	1.003	0.997 – 1.009			
TyG	0.346	2.001	0.473 – 8.456			
eGFR CKD_EPI, ml/min/1.73 m^2^	0.324	0.986	0.960 – 1.014			
Lipid lowering therapy, n	0.302	1.762	0.601 – 5.162			
**Insulin, n**	**0.040**	**3.097**	**1.053 – 9.110**	0.564	1.702	0.280 – 10.357
Metformin, n	0.085	2.660	0.874 – 8.099			
GLP1Ra, n	0.276	1.583	0.693 – 3.617			
SGLT2i, n	0.371	0.660	0.365 – 1.641			
Hypertension, n	0.217	1.955	0.674 – 5.669			
Smoking habit, n	0.187	2.240	0.676 – 7.427			
Liver disease, years	0.246	0.949	0.870 – 1.036			
Type 2 diabetes, years	0.451	1.024	0.963 – 1.088			
Visceral Adiposity Index	0.409	1.015	0.980 – 1.050			

BMI, body mass index ALM/Height^2^, Appendicular Lean Mass Height^2^ index; IPAQ, International Physical Activity Questionnaire; CAP, controlled attenuation parameter; ALT, alanine aminotransferase; AST, aspartate aminotransferase; GGT, gamma-glutamyl transferase; ALP, alkaline phosphatase; INR, international normalized ratio; PLT, platelets; HbA1c, glycated haemoglobin; TyG, Triglycerides Glucose Index; HDL-c, high-density lipoprotein cholesterol; LDL-c, low-density lipoprotein cholesterol; CKD-EPI eGFR, chronic kidney disease epidemiology consortium estimated glomerular filtration rate; GLP1Ra, Glucagon-Like Peptide-1 Receptor Agonist; SGLT2i, Sodium-Glucose Co-Transporter-2 Inhibitor.

Statistically significant variables are highlighted in bold type.

**Figure 2:**
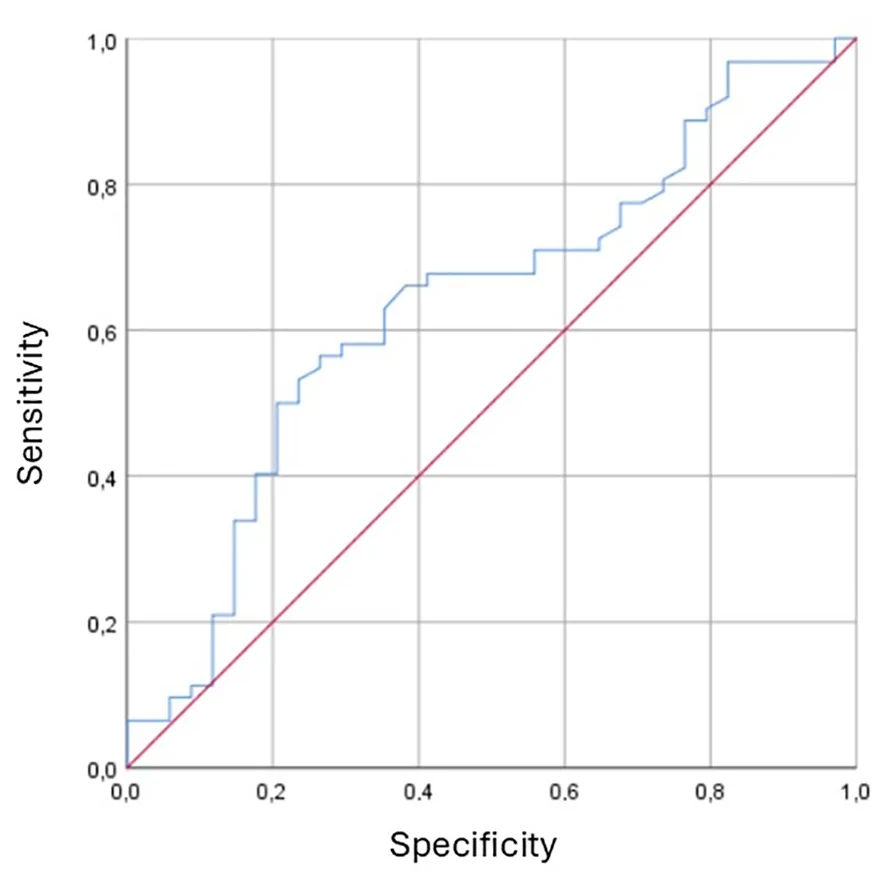
Receiver Operating Characteristic (ROC) curve of fat mass percentage >38.65% for the discrimination of severe hepatic steatosis (CAP ≥280 dB/m). The area under the curve (AUC) was 0.636 (95% C.I. 0.519 – 0.753, p = 0.028). Red diagonal line represents reference line with AUC = 0.5. The x-axis represents the specificity of the test, while the y-axis represents the sensitivity.

ROC curve analysis and the Youden Index were applied to identify specific cut-offs for fat mass percentage and ALM/height^2^ index in detecting severe liver steatosis. A cut-off value of 38.65% was identified for fat mass percentage, with an Area Under the Curve (AUC) of 0.636 (Youden Index = 0.276, 95% C.I. 0.519 – 0.753, p = 0.028), corresponding to a specificity of 64.7% and a sensitivity of 62.9% for the detection of severe steatosis (**Figure 2**). When the conventional sex-specific thresholds (>25% in men and >35% in women) were applied, 97.9% of patients were classified as having pathological adiposity, yielding very high sensitivity (~100%) but extremely low specificity (~3%) for severe steatosis. The cohort-derived cut-off of 38.65% showed a more balanced discriminatory performance, with a sensitivity of 62.9% and a specificity of 64.7%. The same analysis was performed for the ALM/height^2^ index, identifying a cut-off value of 6.7 kg/m^2^; however, this result did not reach statistical significance (AUC = 0.611, 95% C.I. 0.495 – 0.727, Youden Index = 0.245, p = 0.07).

Based on the ROC-derived cut-offs for severe steatosis, the study population was stratified into four groups:

a) patients with neither ALM/height^2^ index <6.7 kg/m^2^ nor fat mass percentage >38.65% (patients without reduced muscle mass and without excess adiposity, n. = 29, 29.89%);
b) patients with both ALM/height^2^ index <6.7 kg/m^2^ and fat mass percentage >38.65% (patients with reduced muscle mass and excess adiposity, n. = 31, 31.95%);
c) patients with ALM/height^2^ index <6.7 kg/m^2^ without fat mass percentage >38.65% (patients with reduced muscle mass, without excess adiposity, n. = 14, 14.43%);
d) patients with fat mass percentage >38.65% without ALM/height^2^ index <6.7 kg/m^2^ (patients without reduced muscle mass with excess adiposity, n. = 23, 23.71%).

The Kruskal Wallis Test demonstrated significant differences in CAP values among the four study groups (H = 15.262, p = 0.002). The highest mean rank (62.28) was observed in participants with high fat mass percentage and ALM/height² ≥6.7 kg/m².

**Figure 3:**
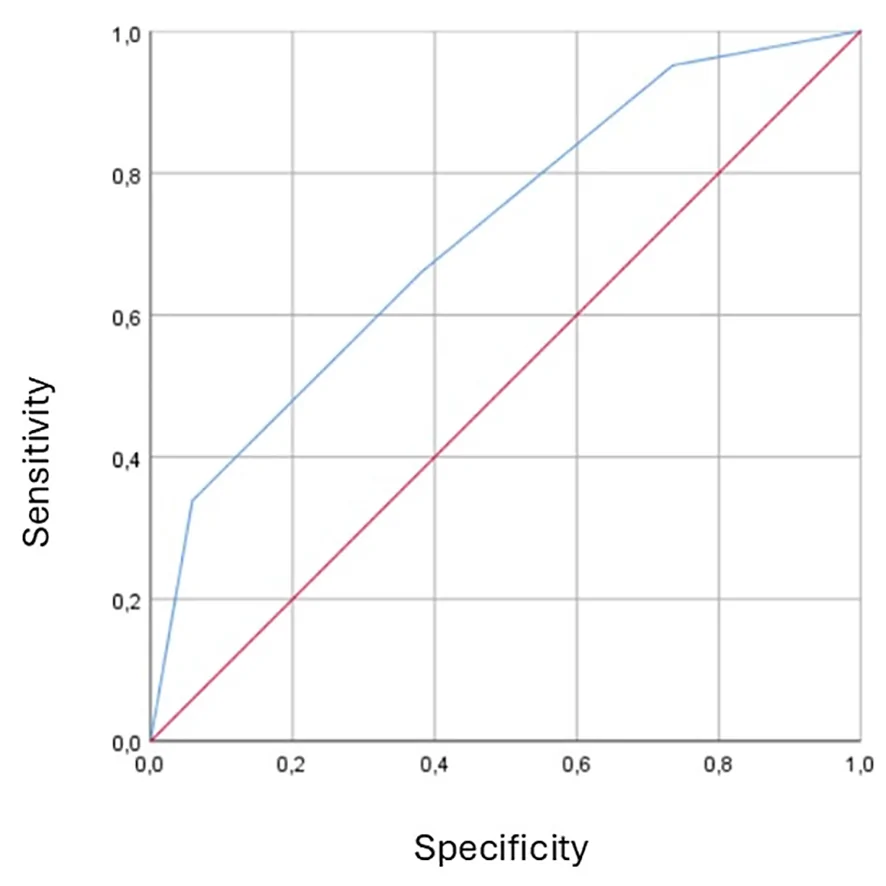
Receiver Operating Characteristic (ROC) curve of the combined model – fat mass percentage >38.65% and appendicular lean mass/height^2^ <6.7 kg/m^2^ – for the discrimination of severe hepatic steatosis (CAP ≥280 dB/m). The area under the curve (AUC) was 0.715 (95% C.I. 0.610 – 0.820, p = 0.001). Red diagonal line represents reference line with AUC = 0.5. The x-axis represents the specificity of the test, while the y-axis represents the sensitivity.

The two ROC-derived cut off values were simultaneously included in a binary logistic regression model for severe steatosis. Predicted probabilities derived from the logistic regression model were saved and subsequently used for a ROC curve analysis to assess the combined discriminative performance of the two body composition parameters. This combined ROC curve comprising both ALM/height^2^ index <6.7 kg/m^2^ and fat mass percentage >38.65% demonstrated a numerically higher discriminative ability for CAP ≥280 dB/m (AUC = 0.715, Youden Index 0.28, 95% C.I. 0.610 – 0.820, p = 0.001; specificity 94.1%, sensibility 40.3%), than each individual body composition parameters (**Figure 3**).

## 4 Discussion

This study examined the associations between body composition, assessed by DXA, and hepatic steatosis severity in a cohort of patients with MASLD and T2D. Overall, the study population presented compensated liver disease – chronic liver disease or liver cirrhosis classified as Child Pugh A – together with a satisfactory glycaemic control, despite high burden of insulin-resistance, as reflected by TyG index values.

A first relevant finding of this study concerns the discrepancy between BMI-based and DXA-based identification of obesity. Specifically, obesity was detected in only 46.4% of patients according to BMI criteria, whereas DXA revealed a pathological fat mass percentage in 97.9% of participants, with a statistically significant difference between the two approaches (p < 0.001). This discordance is consistent with a recognised limitation of BMI as a surrogate measure of adiposity, namely its inability to capture the substantial heterogeneity in body composition among individuals within the same weight category (25). By contrast, waist circumference demonstrated greater concordance with DXA-derived indices of fat distribution, yielding prevalence estimates comparable to those obtained using the pathological android-to-gynoid fat mass ratio (p = 0.28) (26, 27). However, correlation analyses revealed a more nuanced picture: BMI was significantly associated with fat mass percentage (p = 0.001) but not with the android-to-gynoid fat mass ratio (p = 0.964), whereas waist circumference was not significantly correlated with either fat mass percentage (p = 0.460) or fat mass distribution as assessed by the android-to-gynoid fat mass ratio (p = 0.336). Taken together, these findings suggest that neither BMI nor waist circumference alone adequately reflects the complexity of body composition and fat distribution in patients with MASLD and T2D. Direct assessment methods such as DXA may therefore provide complementary information (28–30).

DXA also identified a substantially higher proportion of reduced muscle mass compared with conventional anthropometric measurements. Specifically, reduced appendicular lean mass was detected in 36.1% of participants by DXA, whereas only 20.6% exhibited reduced calf circumference (p = 0.016). These findings suggest that, in patients with obesity and MASLD, calf circumference may be confounded by excess adiposity, thereby limiting its reliability for the early detection of reduced muscle mass (31, 32), and further underscoring the need for more precise tools in the clinical assessment of this population. Muscle mass assessment may be particularly important before weight-loss interventions, including glucagon-like peptide-1 receptor agonists (GLP-1 RAs) or dual GLP-1/glucose-dependent insulinotropic polypeptide (GIP) receptor agonists, because these treatments may be accompanied by loss of lean mass (33, 34). Baseline assessment may help to guide exercise and nutritional interventions intended to preserve muscle mass (35, 36).

Regarding the relationship between body composition parameters and liver disease, regression analyses showed that advanced liver fibrosis was not independently associated with the evaluated variables related to adiposity and reduced muscle mass. Although no associations were observed between BMI and liver stiffness >8 kPa (p = 0.501), waist circumference emerged as an independent correlate of liver fibrosis (p = 0.004), underscoring the role of visceral fat distribution in the metabolic determinants of liver disease progression (37–40). Nonetheless, these findings should be interpreted cautiously, as the relatively small number of patients with cirrhosis may have limited the robustness of the analysis.

Measures of both overall adiposity, including BMI and fat mass percentage, and central adiposity, including WC and VAI, correlated with CAP (37, 40–42). Both indices reflecting overall fat accumulation – such as BMI (p = 0.007) and fat mass percentage (p = 0.016) – and parameters reflecting fat distribution – including waist circumference (p = 0.005) and visceral adiposity index (p = 0.005) – significantly correlated with liver steatosis. In multivariate logistic regression analysis, fat mass percentage remained independently associated with severe steatosis (p = 0.011). This finding supports an association between DXA-derived adiposity measures and hepatic steatosis but does not allow to establish superiority over conventional anthropometric measures. The weak correlations probably reflect the multifactorial pathophysiology of MASLD. Indeed, hepatic steatosis is influenced by multiple metabolic, inflammatory, genetic, and environmental factors, including insulin resistance, alterations in lipid metabolism, genetic predisposition (*e.g. PNPLA3, TM6SF2*, and *MBOAT7* variants), as well as high fructose intake and low physical activity, all contributing to disease onset and severity (43). No significant correlations were observed between CAP and TyG index (Rho = 0.200, p = 0.51), LDL cholesterol (Rho = 0.137, p = 0.182), or HbA1c (Rho = 0.152, p = 0.138). In our cohort, these findings may reflect the adequate lipid and glycaemic control achieved through pharmacological treatment, which may have masked the contribution of these metabolic parameters to the development and progression of hepatic steatosis.

ALM/height² index was negatively correlated with CAP values (Rho = −0.276, p = 0.006) and remained independently associated with severe steatosis at multivariate analysis (p = 0.013). These findings indicate that lower muscle mass is associated with greater hepatic fat content, thereby supporting the concept of a protective role of skeletal muscle against the development and progression of liver steatosis (9, 44, 45). Skeletal muscle is a major site of insulin-mediated glucose uptake and fatty acid oxidation; consequently, its depletion contributes to insulin resistance, increased hepatic free fatty acid influx, and *de novo* lipogenesis (46, 47). Moreover, the relationship appears to be bidirectional, as MASLD itself may promote muscle catabolism through chronic inflammation, altered protein metabolism and systemic metabolic dysfunction (48, 49). Although these data support the inclusion of muscle mass in phenotypic characterisation, they do not establish a protective causal effect because of the cross-sectional study design.

Additional insights emerge from ROC curve analyses. Fat mass percentage demonstrated a modest yet statistically significant ability to discriminate severe steatosis, with an optimal cut-off of 38.65% (AUC of 0.636, sensitivity of 62.9%, specificity of 64.7%), slightly exceeding the threshold proposed by the WHO for the definition of obesity (22). By contrast, ALM/height^2^ alone did not achieve statistical significance (AUC 0.611, p = 0.07), suggesting that reduced muscle mass, although biologically relevant, is insufficient as a stand-alone discriminator of severe steatosis.

The model combining fat mass percentage >38.65% and ALM/height² <6.7 kg/m² demonstrated a numerically higher discriminative ability than either parameter alone (AUC of 0.715, 95% CI 0.610–0.820, p = 0.001), which may improve risk stratification for severe steatosis in patients with MASLD and T2D. The four-group analysis showed the highest CAP values in participants with high fat mass percentage and preserved ALM/height², suggesting that adiposity was the predominant correlate of hepatic fat accumulation in this cohort, while reduced muscle mass exerts a modulating effect (41, 50, 51). This finding aligns with the concept of “sarcopenic obesity”, a condition characterised by the coexistence of excess fat mass and low muscle mass, which has been increasingly recognised as a distinct and particularly high-risk phenotype in MASLD (50, 52, 53). The improved performance of the combined model supports a multifactorial framework for hepatic steatosis, whereby fat accumulation and muscle depletion interact synergistically to influence intrahepatic lipid content (54).

Overall, DXA-derived body composition parameters may complement conventional anthropometric measures by providing a more detailed characterization of metabolic balance in MASLD patients with T2D. Their incremental value for identifying severe hepatic steatosis remains to be confirmed in larger studies that directly compare predictive models.

Several limitations of this study should be acknowledged. First, the sample size, particularly after stratification into the four body composition phenotypes, was relatively small, potentially limiting the statistical power of subgroup analyses. Although the achieved sample size was sufficient to detect moderate discriminative performance, the study may have been underpowered to detect small differences between correlated ROC curves. In addition, the low prevalence of sarcopenia diagnosed by the combination of reduced muscle mass and low handgrip strength didn’t allow this parameter to be included in the statistical analyses, thereby justifying the use of reduced muscle mass alone. Furthermore, the study population was recruited from a single tertiary centre and consisted exclusively of patients with T2D who underwent DXA as a part of osteoporosis screening. These features limit the generalisability of the findings to broader MASLD populations, particularly younger individuals and patients without T2D. Therefore, the DXA-derived cut-offs identified in this cohort require external validation before their clinical application can be recommended, as they were derived from the same cohort and their discriminatory performance may therefore be overestimated. Similarly, the ROC curve analyses should be considered as exploratory and warrant validation in larger independent cohorts, including MASLD patients without T2D. Longitudinal studies are also needed to evaluate changes in body composition during treatment with GLP-1 receptor agonists and Sodium-Glucose Co-Transporter-2 (SGLT2) inhibitors, and their effects on the severity of hepatic steatosis. Finally, the cross-sectional design of the study precludes any inference regarding causal relationship.

## 5 Conclusions

In patients with T2D and MASLD, DXA identified excess adiposity and reduced muscle mass more precisely than conventional anthropometric measures. Hepatic steatosis severity appears to be primarily driven by adiposity, while reduced appendicular lean mass exerts an independent modulating effect, consistent with a protective role of skeletal muscle against intrahepatic lipid accumulation. Neither parameter alone showed adequate discriminative ability for severe steatosis; however, their combination — integrating fat mass percentage and ALM/height² index — significantly improved risk stratification, supporting a multifactorial model in which adiposity and muscle mass interact to influence liver fat content. These findings suggest that DXA-based body composition assessment may improve phenotypic characterization and risk stratification in selected patients with MASLD and T2D, despite larger validation studies are required.

## Data Availability

The raw data supporting the conclusions of this article will be made available by the authors, upon reasonable request.
